# Extravasation of biodegradable microspheres in the rat brain

**DOI:** 10.1080/10717544.2023.2194579

**Published:** 2023-03-30

**Authors:** Anne-Eva van der Wijk, Theodosia Georgakopoulou, Rob Steendam, Johan Zuidema, Peter L. Hordijk, Erik N.T.P. Bakker, Ed van Bavel

**Affiliations:** a Deparment of Biomedical Engineering and Physics, Amsterdam UMC Location University of Amsterdam, Amsterdam, The Netherlands; b Neurovascular Disorders Program, Amsterdam Neuroscience, The Netherlands; c Microcirculation Program, Amsterdam Cardiovascular Sciences, Amsterdam, The Netherlands; d InnoCore Pharmaceuticals, Groningen, The Netherlands; e Deparment of Physiology, Amsterdam UMC Location Vrije Universiteit Amsterdam, Amsterdam, The Netherlands

**Keywords:** Microsphere extravasation, angiophagy, drug delivery, blood-brain barrier, biodegradable polymer

## Abstract

Drug development for neurological diseases is greatly impeded by the presence of the blood-brain barrier (BBB). We and others previously reported on extravasation of micrometer-sized particles from the cerebral microcirculation – across the BBB – into the brain tissue over the course of several weeks. This mechanism could potentially be used for sustained parenchymal drug delivery after extravasation of biodegradable microspheres. As a first step toward this goal, we set out to evaluate the extravasation potential in the rat brain of three classes of biodegradable microspheres with drug-carrying potential, having a median diameter of 13 µm (80% within 8–18 µm) and polyethylene glycol concentrations of 0%, 24% and 36%. Extravasation, capillary recanalization and tissue damage were determined in a rat cerebral microembolization model at day 14 after microsphere injection. Microspheres of all three classes had the potential to extravasate from the vessel into the brain parenchyma, with microspheres without polyethylene glycol extravasating the fastest. Microembolization with biodegradable microspheres led to impaired local capillary perfusion, which was substantially restored after bead extravasation. We did not observe overt tissue damage after microembolization with any microsphere: we found very limited BBB disruption (IgG extravasation), no microgliosis (Iba1 staining) and no large neuronal infarctions (NeuN staining). In conclusion, biodegradable microspheres with different polymer compositions can extravasate into the brain parenchyma while causing minimal tissue damage.

## Introduction

One of the major challenges in the neurological field is the development of therapeutics for brain diseases. In the past decades, clinical trials for treatment of brain disorders ranging from Alzheimer disease (Cummings, 2018) to glioblastoma (Jacus et al., 2016) have led to failure, almost without exception. This is largely due to the presence of the blood-brain barrier (BBB), which is a major hurdle for delivery of drugs from the blood to the brain interstitium, since the BBB only allows for passage of small molecules that are both lipid-soluble and have a molecular weight of <400–500 Da (Pardridge, 2005). This precludes the use of ∼98% of all small molecules, and all large molecule therapeutics (such as monoclonal antibodies, recombinant proteins, gene therapeutics) (Pardridge, 2005). Many attempts have been made to re-engineer drugs in order to circumvent the BBB, *e.g.* through nanocarriers (Beduneau et al., 2007), receptor-mediated transport (Giugliani et al., 2018) or ultrasound-induced BBB disruption (Carpentier et al., 2016), but with limited success. Thus, a drug delivery platform to transport therapeutics across the BBB, where they can exert their effects locally in the brain parenchyma, would be a major step forward in this field.

In previous studies we observed that flow-obstructing, polystyrene particles with diameters of 15 to 50 micrometer that were injected intra-arterially into the rat brain extravasated from the vessel into the brain tissue over the course of several weeks (van der Wijk et al., 2019, 2020). This process was previously observed in the mouse brain, lungs, heart and retina (Lam et al., 2010; Grutzendler et al., 2014), and in the human retina (Cho et al., 2016) and was coined angiophagy (Grutzendler et al., 2014). Extravasation of polystyrene microspheres was associated with restoration of blood flow (Lam et al., 2010; Grutzendler et al., 2014; van der Wijk et al., 2019, 2020) and, importantly, caused only mild and transient tissue damage (van der Wijk et al., 2019, 2020). Of note, these microspheres are several orders of magnitude larger than nanoparticles that are being used in drug delivery studies (Lam et al., 2018) and their extravasation does not require specific endothelial receptor binding, but rather physical entrapment of the particle in the smaller arterioles and capillaries (van der Wijk et al., 2020).

This extravasation mechanism could potentially be exploited to deliver drugs across the BBB into the brain parenchyma, using biodegradable microspheres. Such a microsphere would need to be transported across the endothelium, followed by slow release of the drug. As a first step, we studied the transendothelial migration of several fluorescent biodegradable microspheres, not loaded with drugs.

Biodegradable microspheres are widely used as long-acting injectable (LAI) depot formulations to treat chronic diseases. Typically, LAI microspheres are composed of polymers that release their bioactive cargo over a specific time frame, related to polymer swelling, increasing porosity and hydrolytic degradation. They are usually injected subcutaneously or intramuscularly. The released drugs have local or remote targets (Teekamp et al., 2017; Sandker et al., 2018; Nkanga et al., 2020). Physicochemical properties of copolymer blocks can be varied to adjust drug elution profiles and degradation characteristics. Here, we used poly(ether ester urethane) multi-block copolymers (SynBiosys® (InnoCore Pharmaceuticals, Groningen, The Netherlands) composed of various combinations of lactide, glycolide, ε-caprolactone, dioxanone and polyethylene glycol. The composition of these multi-block copolymers can be customized to meet specific needs regarding their physical characteristics, including degradation, hydrophilicity, swelling degree, erosion kinetics and drug release kinetics. Microspheres prepared of poly(ether ester urethane) multi-block copolymers with different composition have been found suitable for the sustained release of small molecules (Zandstra et al., 2015; Sandker et al., 2018), proteins (Hughes et al., 2021; Blanco-Blázquez et al., 2023), growth factors (Blanco-Blázquez et al., 2023) and monoclonal antibodies (Teekamp et al., 2017; Steendam et al., 2022).

The aim of this study was to evaluate microspheres prepared of poly(ether ester urethane) multi-block copolymers with different composition for their ability to extravasate. This is a first step in evaluating their potential as a drug delivery platform to target the brain in neurological disease. Here, we determined (1) whether fluorescent biodegradable microspheres, injected into the common carotid artery, extravasate through angiophagy from the cerebral microcirculation in rats, (2) whether there is a difference in extravasation speed between biodegradable microspheres of varying composition, and (3) how microembolization with biodegradable microspheres affects brain tissue.

## Materials and methods

### Polymer synthesis and characterization

SynBiosys® multiblock copolymers (InnoCore Pharmaceuticals, The Netherlands) were synthesized and characterized as described before (Stanković et al., 2014). The polymer for microsphere M1, abbreviated as 50GL20L40, consisted of a poly(DL-lactide-co-glycolide) block with a molecular weight (MW) of 2,000 g/mole (GL20) and a poly(DL-lactide) block, MW 4,000 g/mole (L40) in a 50/50 wt.% block ratio. The polymer for M2, abbreviated as 50CP10C20-D25, consisted of a poly(ε-caprolactone)-(polyethylene glycol)-poly(ε-caprolactone) block, MW 2000 g/mole containing 50 wt.% of PEG MW 1000 g/mole (CP10C20) and a poly(*p*-dioxanone) block, MW 2,500 g/mole (D25) in a 50/50 wt.% block ratio. The total PEG fraction of this polymer for M2 was 24 wt.%. The polymer for M3, abbreviated as 50CP30C40-LL40, consisted of a poly(ε-caprolactone)-polyethyleneglycol-poly(ε-caprolactone) block, MW 4000 g/mole containing 75 wt.% of PEG MW 3000 g/mole (CP10C20) and a poly(L-lactide) block, MW 4,000 g/mole (LL40) in a 50/50 wt.% block ratio. The total PEG fraction of this polymer for M3 was 36.4 wt.%.

The chemical composition of the polymers was determined by ^1^H NMR using a Bruker Avance DRX 500 MHz NMR spectrometer (B AV 500) equipped with a Bruker Automatic Sample Changer (BACS 60) (Bruker, Massachusetts, USA) operating at 500 MHz and using samples of 25 mg polymer in 1.3 g deuterated chloroform. The d1 waiting time was set to 20 s, and the number of scans was 16. Spectra were recorded from 0 to 14 ppm. The composition of the polymers as determined by ^1^H NMR is listed in Table 1. Intrinsic viscosity of the polymers was measured using an Ubbelohde viscosimeter (DIN), type 0 C (Si Analytics, Mainz, Germany) supplied with a Si Analytics viscosimeter including a water bath. The measurements were performed in chloroform at 25 °C. The polymer concentration in chloroform was such that the relative viscosity was in the range of 1.2–2.0. The intrinsic viscosity of the polymers are listed in Table 1.

**Table 1.** Chemical composition and properties of polymers used for microspheres M1, M2 and M3.

Polymer	50GL20L40, M1	50CP10C20-D25, M2	50CP30C40-LL40, M3
PEG MW (g/mole)	–	1000	3000
PEG wt.%	0.0	24.0	36.4
Lactide wt.%	71.6	0.0	47.2
Glycolide wt.%	20.3	0.0	0.0
Caprolactone wt.%	0.0	23.3	12.1
Dioxanone wt.%	0.0	45.7	0.0
BDO wt.%	3.2	1.6	1.0
BDI wt.%	4.9	5.4	3.3
IV (dl/g)	0.76	0.73	0.78

PEG: polyethylene glycol; MW: molecular weight; wt%: weight percentage; BDO: 1,4 butanediol; BDI: 1,4-butanediisocyanate. IV: intrinsic viscosity.

### Preparation of microspheres

Microspheres with narrow particle size distributions (M1, M2, M3) were prepared of 50GL20L40, 50CP10C20-D25 and 50CP30C40-LL40 via membrane emulsification using an oil-in-water single emulsion solvent extraction/evaporation method. For the purpose of this study, all microspheres were loaded with 1 wt.% of the fluorescent molecule Lumogen red 305 (BASF Color & Effects GmbH, Ludwigshafen, Germany) to facilitate visualization in the rat brain. Polymers were dissolved in dichloromethane (DCM) to a concentration of 10 w/w% and Lumogen red 305 was added, where after the solution was filtered (PTFE, 0.2 μm). Subsequently, the filtered solution was pumped (5.0 mL/min) through a stainless steel membrane with a pore size of 5 µm into a continuous phase consisting of an aqueous solution containing 0.4% polyvinyl alcohol. The obtained emulsion was collected in a 1 L beaker glass and stirred for 3 h (200 rpm, magnetic stirrer) to evaporate DCM. The hardened microspheres were subsequently washed with Millipore water, collected by filtration (5 μm filter), frozen and lyophilized using a Christ Alpha 2–4 LSC Plus freeze-dryer (Martin Christ Gefriertrocknungsanlagen GmbH, Osterode am Harz, Germany). Vials containing frozen microspheres were placed on pre-cooled shelves (−45 °C), whereafter vacuum was applied. The pressure was gradually reduced to 2 mbar and the microparticles were dried for 3 hrs at a shelf temperature of −10 °C followed by 8 hrs at 20 °C, whereafter the pressure was gradually reduced to 1 mbar over 2 hrs. Final drying was performed at 20 °C and a pressure of 0.001 mbar for 2 hrs.

Dried microspheres were suspended in an aqueous 0.6% sodium carboxymethyl cellulose solution at a concentration of 0.283 mg/mL, representing approximately 200,000 particles per mL, as based on their weight, volume averaged particle size and polymer density. Microsphere suspensions were shipped and stored refrigerated at ∼5 °C until further use. A single production batch was made for each of the three microsphere types.

### Characterization of microspheres

The surface morphology of the microspheres was examined by scanning electron microscopy (SEM) using a Zeiss Gemini Sigma 300 (Göttingen, Germany). Samples of microspheres were adhered to a sample holder using double-sided adhesive carbon conductive tape and sputter coated with a thin layer of gold. Samples were then imaged using a 3 kV electron beam.

Average size and particle size distribution (PSD) of the microspheres was measured by laser diffraction using a Horiba LA-960 Laser Diffraction Particle Analyzer (Horiba, Japan). In brief, for each of the three batches M1, M2 and M3, a suspension of the microspheres in demineralized water was added to a 15 mL measuring cell combined with a stirring magnet to disperse the particles. Microsphere concentration was adjusted to have a transmittance within 70 to 90%. Two light sources were used in the system, one laser with a wavelength of 650 nm and one LED with a wavelength of 405 nm. Together with multiple detectors, the scattering pattern of the particles in solution was determined. Using the Fraunhofer theory, the PSD was calculated within a range of 10 nm–5000 µm. A single fold measurement of 20 seconds was recorded. The coefficient of variation (C.V.) of the particle size distribution was calculated according to Eq. (1).

(1) $$C.V.=\frac{\mathrm{Std}.\mathrm{Dev}.}{D_{v(}50)}$$

Where *D_v_* (50) is the volume-based mean diameter, and Std.Dev. the standard deviation of this diameter. The span of the particle size distribution was calculated using Eq. (2)

(2) $$\mathrm{Span}=\frac{D_{v(}90)-D_{v(}10)}{D_{v(}50)}$$

where D_v_ (10), D_v_ (50) and D_v_ (90) represent volume-based particle sizes for 10%, 50% and 90% undersize, respectively.

### Ethics statement

The experiments in this study were conducted in female and male Wistar rats (21–23 weeks old, Charles River). Rats were housed in pairs under a 12 h light-dark cycle and fed ad libitum with standard laboratory chow and free access to water. All experiments were conducted in accordance with the ARRIVE guidelines and European Union guidelines for the care laboratory animals (Directive 2010/63/EU). The surgical protocols in this study were performed with approval of the local committee on the Ethics of Animal Experiments of the University of Amsterdam, Academic Medical Center (permit number: 18-5484-1-02). All surgical procedures were conducted under isoflurane inhalation anesthesia mixed with oxygen while the body temperature was monitored with a feedback-regulated heating pad.

### Cerebral embolization procedure

Cerebral embolization was done as described previously (van der Wijk et al., 2020) under isoflurane inhalation anesthesia (induction 4%, maintenance 2–2.5% in 1 L/min O_2_) and subcutaneous buprenorphine (0.05 mg/kg) for analgesia. In short, the left common carotid (CCA), the internal carotid (ICA) and external carotid (ECA) artery were exposed and the ECA and occipital artery were temporarily ligated. M1, M2 or M3 Lumogen Red-loaded fluorescent microspheres with a median diameter of 12.7–12.9 µm (see Table 2) were then injected into the CCA using a 29 G insulin needle. Considering their size, these microspheres are expected to lodge in small arterioles and capillaries (Blinder et al., 2013). Based on previous studies (van der Wijk et al., 2019, 2020) and pilot experiments (data not shown) we injected around 40,000 microspheres in a total volume of 200 µl physiological buffer into the common carotid artery of rats to ensure sufficient microsphere infusion and initiation of extravasation, while causing minimal tissue damage. Intra-carotid microsphere injection leads to a stochastic distribution of microspheres throughout the intervention hemisphere, whereas the control hemisphere is not affected, barring an occasional contralateral microsphere close to the midline (Georgakopoulou et al., 2021). The right CCA was not injected, and therefore the right hemisphere served as the untreated control.

**Table 2.** Microsphere characteristics.

Microsphere group	M1	M2	M3
Polymer grade	50GL20L40	50CP10C20-D25	50CP30C40-LL40
Lumogen red 305 content (wt.%)	1.0	1.0	1.0
Particle concentration	40,000 / 200 µl	40,000 / 200 µl	40,000 / 200 µl
D_v_(10)* (µm)	7.8	8.2	7.6
D_v_(50)* (µm)	12.7	12.9	12.7
D_v_(90)* (µm)	18.6	18.2	18.8
SPAN	0.85	0.77	0.88
CV (%)	32.7%	29.6%	33.7%

* D_v_(10), D_v_(50), and D_v_(90) represent volume-based particle sizes for 10%, 50%, and 90% undersize, respectively.

### Tissue preparation and immunofluorescence staining

Animals were killed on day (D) 14 (*n* = 6–8 per group) after surgery. After induction of anesthesia, the vasculature was labeled by an intravenous injection of DyLight 488 labeled tomato lectin (1 mg/kg; DyLight 488 labeled lycopersicon Esculentum tomato, Vector Laboratories, Burlingame, CA), which was allowed to circulate for 5 min. Rats were given 100 µl heparin i.p. and after increasing isoflurane to 5% animals were transcardially perfused with heparinized PBS followed by tissue fixation with 4% paraformaldehyde at 80 mmHg. The brain was removed and post-fixed in 4% paraformaldehyde overnight and then stored in 30% sucrose at 4 °C for approximately two days. Coronal free-floating sections of the cerebrum were cut using a cryomicrotome (Leica Microm HM 560 M, Nussloch, Germany) at 100-μm thickness. Sections were stored serially in tissue collection suspension (containing 30% sucrose and 30% ethylene glycol in 0.1 M phosphate buffer) at −20 °C. Tissue preparation and immunofluorescence staining was done as described previously (van der Wijk et al., 2020), with the following adjustments: blocking buffer contained 5% normal goat serum and 0.1% Triton X-100 in PBS. The following antibodies were used: (primary) rabbit polyclonal anti-laminin antibody (diluted 1:500, Cat #L9393; Sigma-Aldrich, Zwijndrecht, The Netherlands), rabbit polyclonal anti-Iba 1 antibody (diluted 1:1000, Cat #019-19741, Wako, Neuss, Germany), mouse anti-NeuN antibody (NEUronal Nuclei, clone A60; diluted 1:200, Cat #MAB377, Millipore BV, Amsterdam, The Netherlands), and (secondary) goat-anti-mouse Cy5 (diluted 1:500) or goat-anti-rabbit Cy5; diluted 1:200) diluted in blocking buffer. The IgG staining was done by incubating brain sections with a goat anti-rat-IgG conjugated to Cy5 (diluted 1:250, Cat #A21208, ThermoFisher, Landsmeer, The Netherlands) overnight at room temperature. Specificity of the staining was checked by excluding the primary antibody.

Images of 100 µm-thick brain sections were captured using a confocal laser scanning microscope SP8-X DLS Lightsheet (Leica Microsystems, Wetzlar, Germany) with a 10 × 0.5 NA (air) objective for Iba1, NeuN and IgG, or SP8-X (Leica Microsystems) with a 20 × 0.75 NA (oil) objective for laminin. We used relatively thick session for confocal imaging in order to better image the spatial relation between the microvessels and the 13 micron microspheres, needed for determining the extravasation state, and to include the (oblique) vessels over a sufficient length to allow assessment of perfusion state. In order to cover the full thickness, z-stacks were constructed of 5 micron optical slice thickness and a maximum intensity projection was made over the 100 micron. Signal intensity decreased in deeper layers but was strong enough to allow a sufficient signal to noise ratio and visualization. For the imaging and quantification of IgG leakage and Iba1 signal intensity, we used a single 5 micron plane close to the surface, tiled over both hemispheres.

### Image analysis

Quantification of microsphere extravasation was performed on sections stained for laminin. Microsphere extravasation was scored as ‘in’, ‘going out’ or ‘out’ for at least 30 microspheres per rat (Table S1), based on their position in laminin and lectin images (van der Wijk et al., 2020). From these data an extravasation score was calculated for each animal, where microspheres scored as ‘in’ were assigned 0, ‘going out’ 1 and ‘out’ 2; *i.e.* a higher extravasation score indicates a higher rate of microsphere extravasation. Vessel perfusion was quantified in the same samples, and a vessel was identified as ‘nonperfused’ when there was no lectin distal and/or proximal from the occluding microsphere.

IgG leakage, Iba1 signal intensity and microsphere density were quantified from a single coronal section per animal, covering both hemispheres, constructed by tiling of confocal images. Mean intensity for IgG and Iba1 was calculated over both hemispheres, excluding the ventricles using threshold analysis in FIJI (version ImageJ 1.53c, https://github.com/fiji/fiji).

All analyses on animal brain sections were quantified in a blinded fashion. Total *n* of animals per group are indicated in the figure legends.

### Statistics

Data are depicted as median ± interquartile range (IQR; whiskers represent min – max), and *n* represents the number of animals unless otherwise indicated. Data was tested for normality using a QQ-plot and a Shapiro-Wilk test and depending on the outcome, a parametric or nonparametric test was performed. Differences between groups (M1, M2 and M3) were determined using Kruskal-Wallis with Dunn’s test for multiple comparisons, or by analysis of variance (ANOVA) followed by Tukey Kramer’s test for multiple comparisons. Association of the categorical variables ‘Vessel perfusion’ (yes/no) and ‘Extravasation status’ (in/going out/out) was tested with a Chi-square test for trend. Differences between intervention and contralateral hemispheres were determined using a Wilcoxon signed rank or paired t test. Sex differences were evaluated stratified by M1, M2 and M3 with an ordinary 2-way ANOVA with the factors ‘Sex’ and ‘Microsphere’, and pooled for M1, M2 and M3 per sex with an unpaired t test. Differences were considered statistically significant when *P* ≤ 0.05. Statistical analyses and graphing were performed using GraphPad Prism 9.1.0 software (GraphPad Software, La Jolla, CA).

## Results

### Microsphere injection in rats

22 animals were used in this study. One animal was excluded from all analyses because microsphere injection failed due to extensive bleeding after the intra-arterial injection. In one animal the lectin staining failed. In that animal we could not determine the extravasation status but the animal could be included in the other analyses. All animals survived the surgery and injection of microspheres for the planned period of 14 days without signs of discomfort. All animals showed normal behavior after recovery from surgery, including lack of stroke signs such as circling behavior or hemiparesis. Animals were alert and had normal grooming behavior. Final weight was 466 (IQR 454–484) gram (males, *n* = 10) and 249 (IQR 241–262) gram (females, *n* = 11) at week 20.0 (IQR 19.0–20.7).

### In vitro microspheres size distribution and characteristics

Scanning electron microscopy (Figure S1A) showed that all microspheres were spherical and had a smooth (M1, M2) or somewhat rawer (M3) non-porous surface morphology. Figure S1B shows the particle size distributions of the three microsphere groups as determined by laser diffraction. The volume average particle sizes, d_v_ (50), of the three groups were almost identical (12.7–12.9 μm) (Table 2) as were the values for the coefficient of variance (C.V. 29.6–33.7%) and span (0.77–0.88).

**Figure 1.**
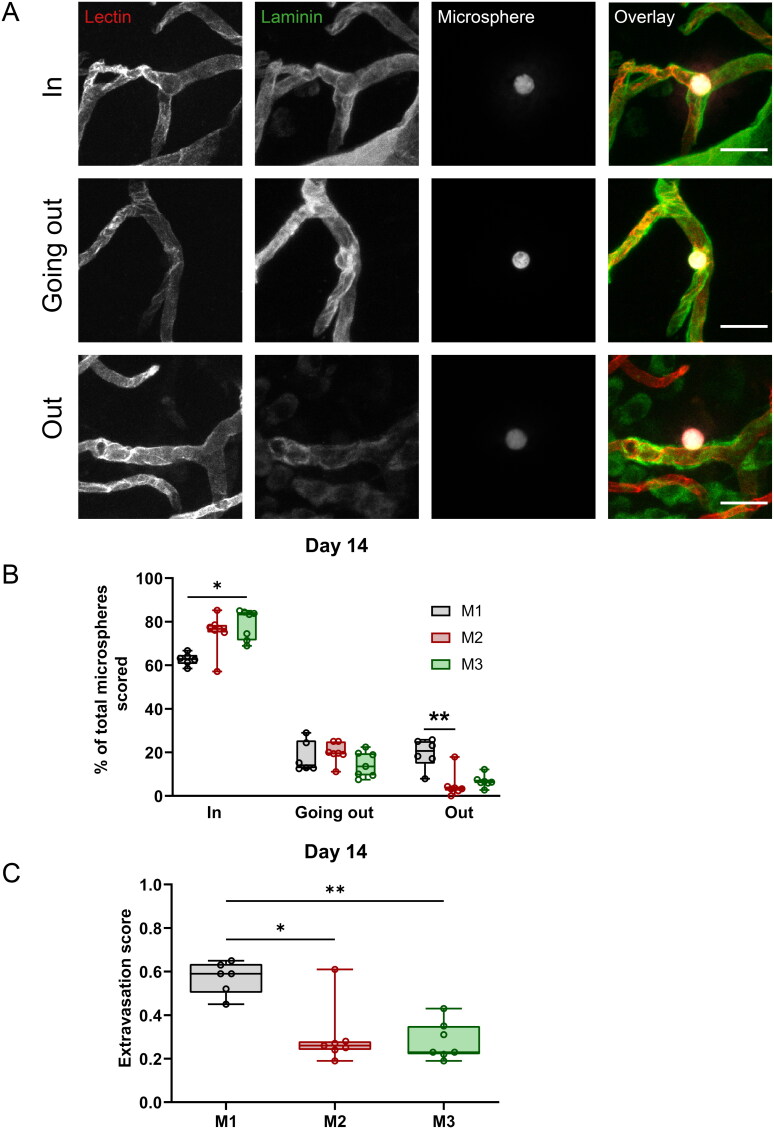
Extravasation of biodegradable microspheres at D14. (A) Examples of biodegradable microspheres (white in overlay) scored as ‘in’, ‘going out’ and ‘out’ in coronal brain sections stained by i.v. injected lectin (red) before killing, and postmortem for laminin (green). Scale bar = 25 µm. (B) Quantification of microsphere extravasation of M1 (black), M2 (red), and M3 (green) microspheres at D14. (C) An extravasation score between 0 and 2 was calculated per rat (‘in’ was weighed as 0, ‘going out’ as 1 and ‘out’ as 2). (B) and (C) M1: *n* = 6, M2: *n* = 7, M3: *n* = 7 animals (one M2 animal was excluded from the extravasation analysis because the i.v. lectin injection failed). Data are depicted as median ± IQR (box) and min – max (whiskers), each data point represents an individual animal. *P < 0.05, **P < 0.01, Kruskal-Wallis with Dunn’s multiple comparison test.

### Extravasation of biodegradable microspheres from the cerebral microcirculation

Part of the biodegradable microspheres from all three groups extravasated from the vessel into the brain at D14 post-injection (Figure 1A,B). Figure 1(A) shows examples of three stages of microsphere extravasation. Extravasation status was assessed based on i.v. lectin injection before killing the animal to visualize the lumen of still perfused vessels, and postmortem staining for the extracellular matrix protein laminin, which clearly outlines the vessel wall (van der Wijk et al., 2020). A median density of 7.6 (IQR 4.8–10.0, *n* = 21) microspheres per mm^3^ was found to lodge in the brain. We classified microsphere extravasation for a median of 40 (IQR 34–55, *n* = 20) microspheres per animal, covering 6.4 mm^3^ (IQR 4.4–8.2, *n* = 20) of brain volume per animal. These data were similar (P = NS) for the three types of microspheres. Figure S2 shows the microsphere densities.

**Figure 2.**
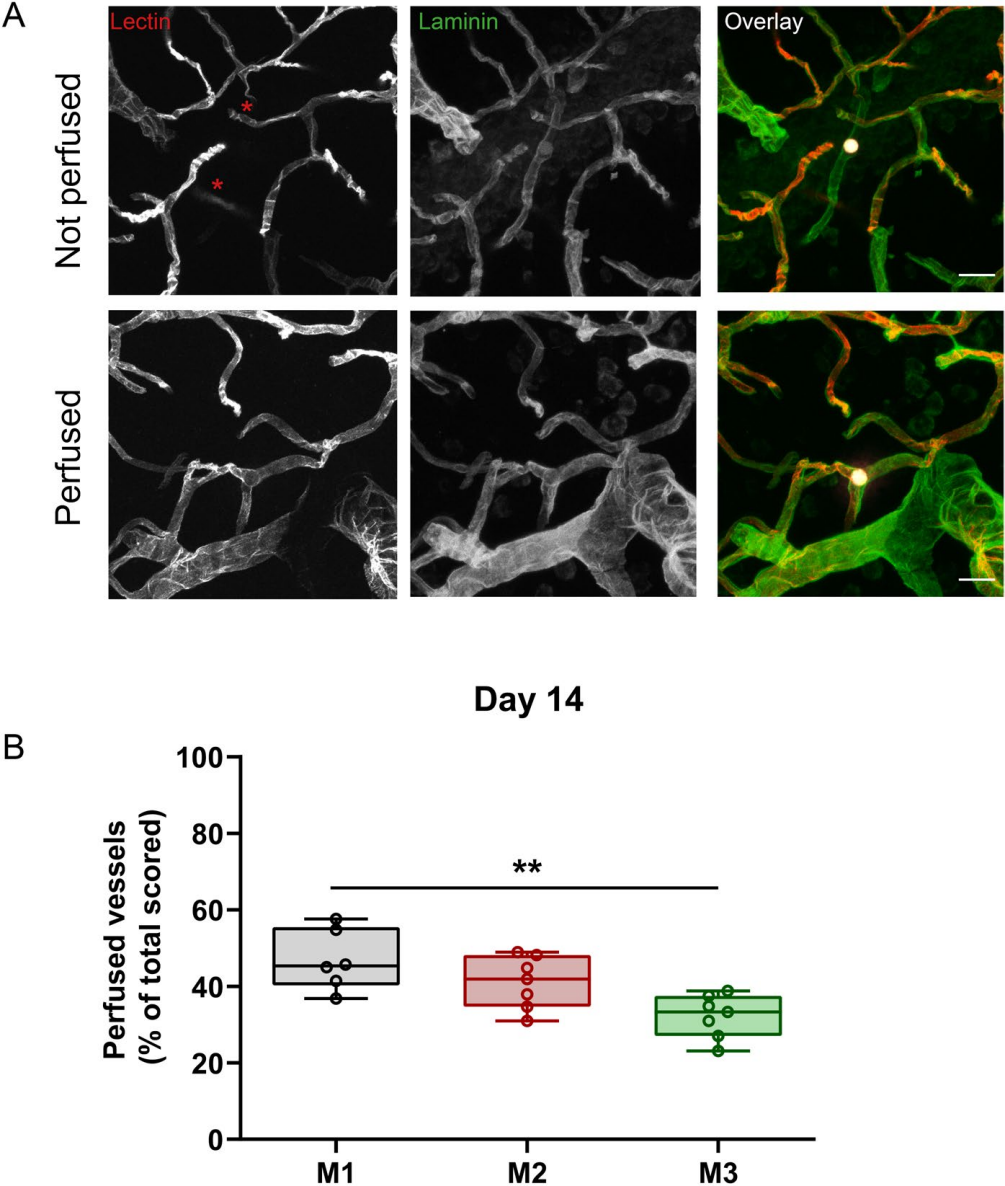
Perfused vessels after microembolization with biodegradable microspheres at D14. (A) Examples of vessels obstructed with biodegradable microspheres. Top panel shows a nonperfused vessel, indicated by lack of i.v. lectin perfusion (red; indicated by asterisks) proximally and distally of the microsphere (white), while the vessel wall is clearly outlined by laminin (green). Bottom panel shows a perfused vessel, despite the presence of a microsphere. Scale bar = 25 µm. (B) Quantification of vessel perfusion after microembolization with M1 (black), M2 (red), and M3 (green) microspheres at D14. M1: *n* = 6, M2: *n* = 7, M3: *n* = 7 animals (one M2 animal was excluded from the perfusion analysis because the i.v. lectin injection failed). Data are depicted as median ± IQR (box) and min – max (whiskers), each data point represents an individual animal. **P < 0.01, ANOVA with Tukey’s multiple comparison test.

At D14, the majority of microspheres was still located inside the vessels (‘in’ or intraluminal), yet there was also a significant portion of microspheres either in the process of extravasation (‘going out’ or extraluminal) or already extravasated (‘out’ or parenchymal; Figure 1(B), Tables S1 and S2). The extravasation score of the group that received M1 microspheres (0.59, IQR 0.50–0.64) was significantly higher compared to the group that received M2 (0.26, IQR 0.24–0.28; *P* = 0.034 M1 vs. M2) or M3 microspheres (0.23, IQR 0.22–0.35; *P* = 0.0090 M1 vs. M3), indicating that the M1 microspheres had the highest extravasation capacity at D14 (Figure 1C).

### Association of vessel perfusion with extravasation status

Microembolization of the brain inevitably leads to perfusion deficits of the obstructed microvessels. We used i.v. injected lectin as a proxy for tissue perfusion in the rat brain, and observed that the majority of obstructed vessels lacked lectin staining either proximally, distally or on both sides of the blocking microsphere (Figure 2A). In some cases, however, there was lectin perfusion despite the presence of an obstruction (Figure 2A). Vessel perfusion was often restored when microspheres were extraluminal or parenchymal. Figure 2(B) shows the quantification of vessel perfusion of all the scored microsphere-containing vessels, *i.e.* irrespective of extravasation status. The M1 group had the most perfused vessels (45.4%, IQR 40.3–55.5%), compared to M2 (41.9%, IQR 34.7–48.2%; *P* = 0.2951, n.s., M1 vs. M2) and M3 (33.3%, IQR 27.0–37.5%; *P* = 0.0032, M1 vs. M3; *P* = 0.0644, n.s., M2 vs. M3). We categorized the scored microspheres according to extravasation (in/going out/out) and perfusion status (yes/no), and found that the two parameters were strongly associated variables for all three microsphere groups (Table S3). This indicates that extravasation of microspheres lead to restoration of vessel perfusion.

### BBB integrity following biodegradable microsphere trapping

**Figure 3.**
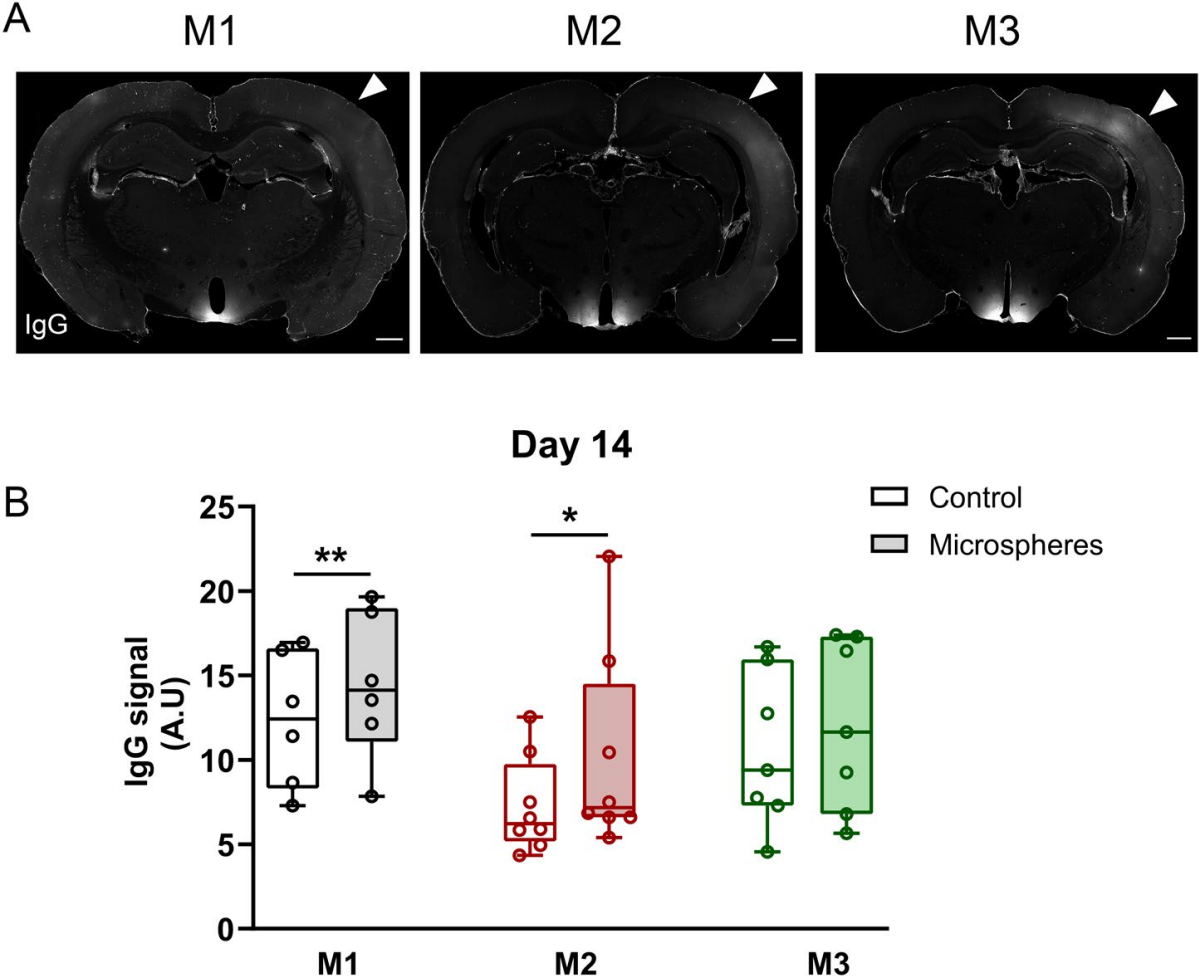
Biodegradable microspheres induce very limited BBB opening at D14. (A) Representative overview images of endogenous IgG staining (white) in coronal sections of a rat brain injected with M1 (black), M2 (red), and M3 (green) microspheres at D14. The IgG signal was very low in both hemispheres (arrowhead indicates intervention side; brightness was increased equally in all images to aid visualization). Scale bar = 1 mm. (B) Quantification of IgG signal intensity in the control (open boxes) and intervention (filled boxes) hemispheres after microembolization with M1 (black), M2 (red) and M3 (green) microspheres at D14. M1: *n* = 6, M2: *n* = 8, M3: *n* = 7 animals. Data are depicted as median ± IQR (box) and min – max (whiskers), each data point represents an individual animal. **P < 0.01, paired t test, *P < 0.05, Wilcoxon signed rank.

The BBB is crucial to maintain tissue homeostasis in physiological conditions (Abbott et al., 2010). To assess whether microembolization with biodegradable microspheres affects BBB function, we stained the rat brain for endogenous IgG after flushing all the blood from the vessels during perfusion-fixation (Figure 3A). This revealed a small but significant increase in IgG staining at day 14 in the embolized hemisphere as compared to the control hemisphere for groups that received M1 and M2 microspheres (Figure 3B).

### Tissue damage following biodegradable microsphere trapping

**Figure 4.**
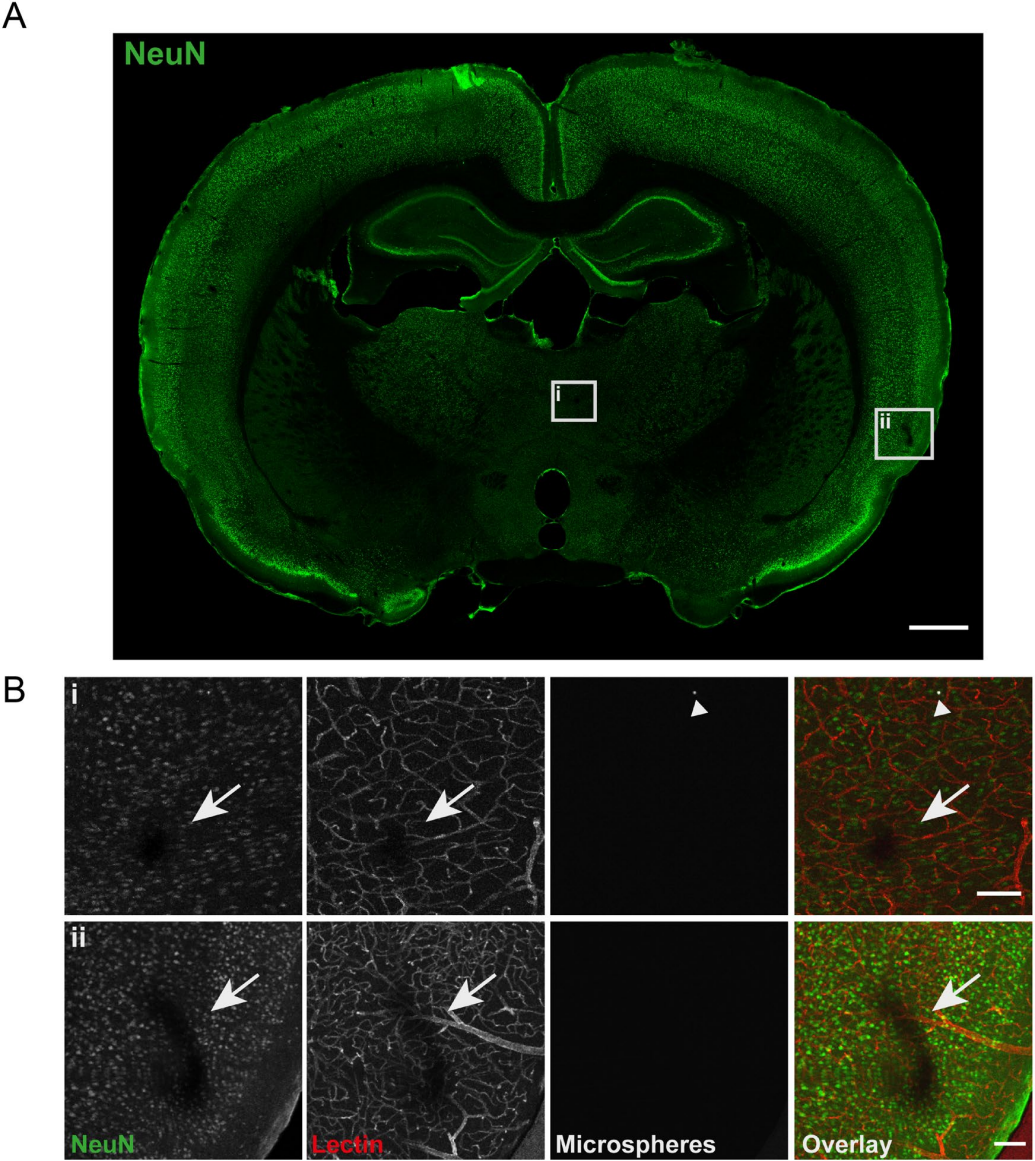
Biodegradable microspheres do not induce overt neuronal damage. (A) Overview image of a coronal brain section stained for the pan-neuronal marker NeuN (green). Two insets show minor infarcted areas in the intervention side, enlarged in (B). This image is from an animal injected with M3 microspheres. Scale bar = 1 mm. (B) Enlarged images of the minor infarcts from (A). i: Infarcted region in the striatum, accompanied by local nonperfusion (red; lack of lectin perfusion), with a microsphere in the vicinity (white; arrow head). ii: Infarcted region in the cortex, accompanied by local nonperfusion, but no microsphere in the near vicinity (in this brain section). This is an example of one of the larger infarcts found, yet it is still relatively small. Scale bar is 100 µm for both images. Arrow: infarcted region, arrowhead: microsphere.

**Figure 5.**
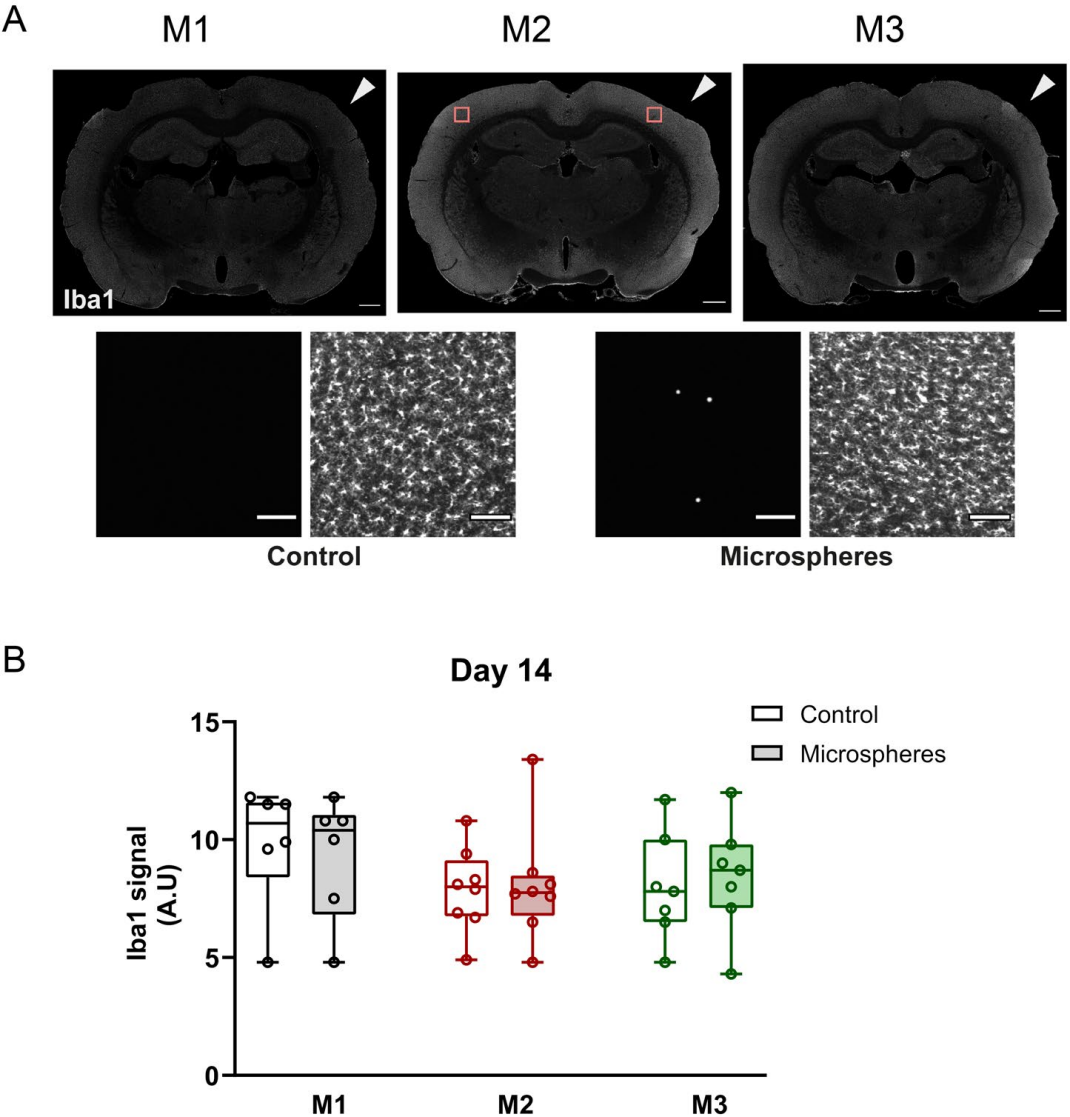
Biodegradable microspheres do not induce microgliosis at D14. (A) Representative overview images of Iba1 staining (marker of microglia; white) in coronal sections of a rat brain injected with M1 (black), M2 (red), and M3 (green) microspheres at D14. Iba1 staining was similar in both hemispheres (arrowhead indicates intervention side; brightness was increased equally in all images to aid visualization). Scale bar = 1 mm. Lower panel images are an enlargement of the boxed regions of the M2 coronal section and show microglia with a quiescent (ramified) morphology in the cortex of the control and intervention hemisphere, also in the near vicinity of microspheres (white). Scale bar is 100 µm. (B) Quantification of the Iba1 signal intensity in the control (open boxes) and intervention (filled boxes) hemispheres after microembolization with M1 (black), M2 (red) and M3 (green) microspheres at D14. M1: *n* = 6, M2: *n* = 8, M3: *n* = 7 animals. Data are depicted as median ± IQR (box) and min – max (whiskers), each data point represents an individual animal.

To determine whether microembolization with biodegradable microspheres of around 13 µm in diameter could be done without causing brain infarctions, we stained the rat brains for NeuN, a neuronal marker (Figure 4A). No large neuronal infarctions were observed in any of the animals after microembolization in all three groups. 19 of the 21 animals did have some micro-infarcts: we found 0 to 5 (median: 2) of such small neuronal micro-infarctions over a full coronal brain cross-section in each animal. The micro-infarcts were generally found in the cortex, presenting as a small region where NeuN signal was absent. In 39 of 53 observed cases the micro-infarct was accompanied by a nonperfused region. In 38 of 53 observed cases, a microsphere was found within 500 µm from the center of the micro-infarct (Figure 4B). The micro-infarcts had median sizes of 74 (53–102, *n* = 21 micro-infarcts in total), 97 (63–169, *n* = 15) and 75 (59–105, *n* = 17) µm along the longest axis for M1, M2 and M3 spheres, respectively (*P* = 0.383, n.s., M1 vs M2; *P* > 0.99, n.s., M1 vs M3 and M2 vs M3). In addition to the limited neuronal damage, we did not observe microgliosis in these animals, as shown by staining for the microglial marker Iba1 (Figure 5A). Microglia had a quiescent morphology (Figure 5A, enlarged images in lower panel) and Iba1 signal was similar in the intervention hemisphere and the control hemisphere in all three groups (Figure 5B). Taken together, these data suggest that microembolization with biodegradable microspheres did not cause major tissue damage in the rat brain in the first 14 days.

### Effect of sex on outcome after brain microembolization

We used two-way ANOVA with ‘Sex’ and ‘Microsphere’ as independent variables to evaluate whether sex affects the outcome of extravasation score, vessel perfusion, IgG and Iba1 signal intensity in the intervention hemisphere for animals stratified as groups that received M1, M2 or M3 microspheres. For both extravasation score (Figure S3A) and vessel perfusion (Figure S3B), there was no effect of sex, nor was there an interaction between sex and microsphere determining these parameters. For IgG (Figure S4A) and Iba1 signal intensity (Figure S4B) in the intervention hemisphere, there was no main effect of microspheres and sex, nor was there an interaction effect. Since the groups are rather small when stratified by microsphere class, we also pooled the microsphere classes to assess whether there is a difference between male and female rats – this was not the case for either of the outcome variables (Figure S3C–D, Figure S4C–D). Together, these data suggest that sex did not affect extravasation, vessel perfusion or effects on the brain tissue after microembolization with biodegradable microspheres.

## Discussion

Currently, there are very few options available to locally treat neurodegenerative diseases such as Alzheimer’s disease, mental disorders or brain tumors, or to apply adjunctive therapy in acute ischemic stroke patients. Progress to treat neurological disease is hampered by the BBB, which blocks entry of almost all therapeutics after systemic delivery (Pardridge, 2005). This is illustrated by aducanumab (U.S. Food & Drug Administration \(FDA\), 2021), a human monoclonal antibody that is administered through i.v. infusions and that selectively reacts with amyloid-β (Aβ) aggregates. In patients, aducanumab reduced Aβ plaques. However, the brain:plasma concentration ratio, although higher than previously reported for other systemically delivered Aβ antibodies (<0.1% brain penetration, (Levites et al., 2006)) was still only 1.3% (Sevigny et al., 2016), meaning that very high systemic doses are needed to attain any clinical effect. Thus, there is still much room for improvement in drug delivery.

In the present study, we studied extravasation of biodegradable microspheres that might have potential to be exploited for drug delivery into the brain. To this end, microspheres need to be administered intra-arterially, which is invasive. Moreover, microembolization of the brain is potentially harmful and may lead to tissue damage. Clearly, these safety concerns should be evaluated. We performed pilot experiments with polystyrene microspheres in the rat brain to assess the optimal number of microspheres injected for particle extravasation, while limiting adverse effects to the brain. For these numbers of injected microspheres, we observed minimal tissue damage after embolization with biodegradable microspheres of all three classes. There was virtually no sign of IgG leakage in the embolized hemisphere, which indicates that the BBB was largely intact at D14 after embolization, although we cannot exclude transient opening at earlier time points (van der Wijk et al., 2020). We found some small neuronal micro-infarctions, mostly localized to the cortical layers (Figure 4). It has been reported in rats that cortical micro-infarcts consequential to single vessel occlusions of penetrating arterioles may result in cognitive dysfunction (Shih et al., 2013), however the median size of micro-infarcts found in the present study was about six times smaller compared to that study. There were also no signs of microgliosis in the intervention side of animals, suggesting absence of ongoing inflammation. Taken together, microembolization of the rat brain with biodegradable microspheres sized ∼13 µm appears to be feasible without overt tissue damage, although some localized damage may occur. Future studies should include *in vivo* imaging (*e.g.* MRI), behavioral and cognitive tests and extensive physiological monitoring to further evaluate tolerability and safety of the procedure.

The current procedure requires intra-arterial application, which was done by common carotid artery injection. This is not a realistic procedure for humans, but here catheterization of the common femoral artery or radial artery would be a feasible although invasive procedure. Such a catheterization procedure is commonly done for coronary artery diagnostics and stenting as well as for acute treatment in ischemic stroke and subarachnoid hemorrhage. Advancing micro-catheters toward distal branches such as the Middle Cerebral Artery M2 or M3 branch would allow for relatively local application (Ospel & Goyal, 2021).

The slow extravasation of drug-loaded microspheres poses a major pharmacokinetic challenge: drug release should be slow enough to avoid intracapillary release, while release after transmigration should allow the build-up of sufficiently high concentrations without overloading capillaries with microspheres. It was not the purpose of this paper to test whether a therapeutic strategy is feasible despite this challenge. Speculatively, higher order drug release kinetics, high affinity drugs, speeding up the extravasation process by further optimization of the polymer design or the use of cargo such as nucleotides or even viruses may provide solutions here. However, as a next step in animals, the ability of the microspheres to transport passive cargo such as fluorescing dyes should be tested first.

Intra-arterial application of drug-loaded microspheres in animal models or in humans has to the best of our knowledge not been studied in the brain. The procedure is not unprecedented in other organs: trans-arterial chemoembolization with microspheres is the gold standard to treat intermediate stage hepatocellular carcinoma (Lencioni et al., 2013). This method is well suited for local delivery of chemotherapeutic drugs at the site of the tumor, while at the same time depriving the tumor’s blood supply through arterial obstruction. This is accomplished by microspheres that are much larger (∼100–300 µm), which elute their content into the vessel lumen at the site of the occluded vessel, from where it diffuses into the liver parenchyma (Namur et al., 2010). While this is different from the current strategy of relying on microsphere extravasation, it illustrates a clear case where the disadvantage of intra-arterial administration is acceptable considering the severity of the disease and treatment outcome. Previous studies have characterized angiophagy as an active and transient process of endothelial cell cytoskeletal remodeling, resulting in local reperfusion through a seemingly normal vessel with intact barrier function (van der Wijk et al., 2019, 2020), rather than a destructive process where emboli end up in the parenchym due to vessel breakdown. While this provides hope for development of a carefully controlled delivery strategy, it clearly remains to be established for which specific neurological diseases, if any, the current strategy could be realistic.

We found that microembolization with biodegradable microspheres led to vessel obstruction and consequent nonperfusion in a large percentage of obstructed vessels. Vessel perfusion was strongly associated with angiophagy, and given more time, restoration of blood flow may take place when a larger portion of microspheres have extravasated into the brain parenchyma (van der Wijk et al., 2019). Based on our previous studies on this Wistar rat model up to days 7 and at day 28 (Lam et al., 2010; Grutzendler et al., 2014; van der Wijk et al., 2019, 2020), we had expected that the majority of injected biodegradable microspheres would have been cleared from the vessels at D14 post-surgery. This was however not the case, as in the present study only ∼20–35% of microspheres (M1, M2 or M3) was in the process of extravasation, or had extravasated at D14. In contrast, about 50–80% of polystyrene microspheres had cleared from the microvessels at D7 (van der Wijk et al., 2019, 2020), and virtually all had undergone angiophagy at D28 after microembolization (van der Wijk et al., 2019). These data suggest that microsphere (polymer) composition may have an effect on extravasation capacity, in line with the fact that we also observed differences between M1, M2 and M3 microspheres.

We prepared microspheres of multiblock copolymers containing different weight fractions and molecular weight of polyethylene glycol. By incorporating increasing amounts of hydrophilic polyethylene glycol (PEG) and PEG segments of increasing molecular weight, the swelling degree and water absorption capacity of multiblock copolymers increases (Bezemer et al., 1999); M1 microspheres were composed of a lactide/glycolide based multiblock copolymer without any PEG that hardly swells under aqueous conditions and are therefore most suited for delivery of small molecules and peptides (Lockwood et al., 2010), whereas M2 (24 wt.% PEG1000) and M3 (36.4 wt.% PEG3000) microspheres, due to the higher swelling degree of their polymers, are suited for delivery of larger molecules, such as proteins (Hughes et al., 2021; Steendam et al., 2022). Although all tested microspheres had the potential to be transported over the BBB, we found that M1 microspheres showed the largest extravasation capacity and highest reperfusion rate at D14. It remains to be established which properties determine the rate of extravasation. The rapidly extravasating polystyrene microspheres in our previous studies are very hydrophobic. Possibly, the water uptake by M2 and M3 microspheres impairs their extravasation due to the increased hydrophilicity, but this needs further study.

It is becoming increasingly recognized that sex differences in pathogenesis, progression and symptoms of disease are strongly understudied in the neuroscience field (or almost any biomedical field for that matter). (Becker et al., 2016; Mazure & Swendsen, 2016). Yet, we did not find significant effects of sex on extravasation, vessel perfusion or effects on the brain tissue after microembolization with biodegradable microspheres, justifying the pooling of data from male and female rats.

### Limitations of the study

In the present study, we took the first steps in determining potential safety hazards of microembolization in the brain. While all animals survived the procedure and the 14 days follow up time without clear signs of distress or cerebral dysfunction, we did not observe the animals for longer periods and it remains to be established whether long-term consequences of microspheres still trapped in the lumen could occur. We also did not study the effect of microembolization on cerebral or systemic safety parameters such as EEG alterations, markers for tissue damage or inflammation, and cardiac function. A more extensive safety profile should be made in future studies, *e.g.* to assess whether the micro-infarcts that we observed in the present study are benign or, despite their minute size, affect cognition and behavior in these animals. We found that a large percentage of biodegradable microspheres was still confined within the microvasculature at D14. Transport of biodegradable microspheres across the BBB and cargo unloading in the brain parenchyma is needed in order to exert truly local effects of therapeutics in the brain. Longitudinal data on microsphere extravasation of all classes would be needed to further develop biodegradable microspheres as a drug delivery platform, *e.g.* to determine optimal timing of drug release and polymer resorption, and hence copolymer block composition. Clearly, further evaluation should demonstrate presence of sustained drug effects in animal models of brain diseases.

We did not study the cellular mechanisms of microsphere extravasation. Our previous work on polystyrene spheres has revealed involvement of endothelial actin cytoskeleton remodeling (van der Wijk et al., 2019, 2020). A better understanding of these mechanisms could help optimizing extravasation by adjusting polymer composition or even by simultaneously targeting endothelial actin dynamics. While we demonstrated extravasation of the microspheres, we did not address release and transport of their cargo in the parenchyma, let alone the biological effectiveness of released compounds. Finally, it remains to be seen to what extent current and future rat studies on biodegradable microsphere extravasation are translatable to humans. This will require a better understanding of the molecular and cellular processes in relation to the differences in the blood-brain barrier between rodents and humans.

## Supplementary Material

Supplemental Material

## Conclusions

In conclusion, we show here that biodegradable microspheres composed of different multiblock copolymers have the potential to extravasate from the cerebral microcirculation into the brain parenchyma. The use of sufficiently small microspheres limited tissue damage due to microembolization. The observed differences in extravasation capacity between the three microsphere compositions warrant further optimization toward the use of such microspheres as a possible drug delivery platform for the sustained release of small molecules, proteins and antibodies to the brain in future studies.
