# Supplementary material for: Extravasation of biodegradable microspheres in the rat brain
Source: Drug Deliv. 2023 Mar 30;30(1):2194579. doi: 10.1080/10717544.2023.2194579 (PMC10064830; doi:10.1080/10717544.2023.2194579)
Supplement: Supplemental Material [file IDRD_A_2194579_SM0599.docx]

## Supplement van der Wijk et al. Biodegradable microsphere extravasation


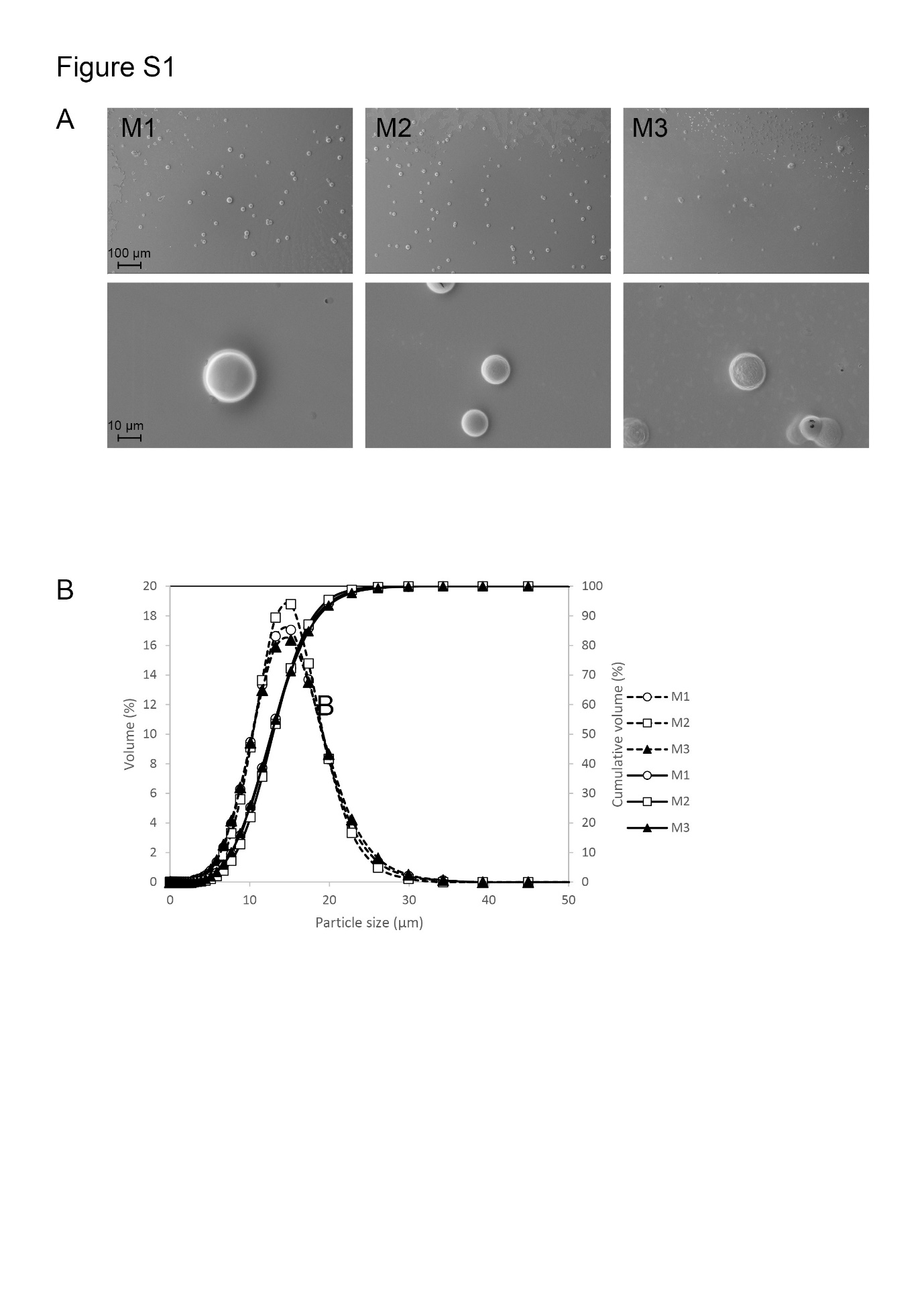


*Figure S1:* **A)** SEM images of M1, M2 and M3 show a round shape of these microspheres, with smooth surface for M1 and M2 and a somewhat rougher surface for M3. **B)** Particle size distribution of microspheres M1, M2 and M3 with volume percentage on the left axis (dashed lines) and cumulative volume percentage on the right axis (solid lines).


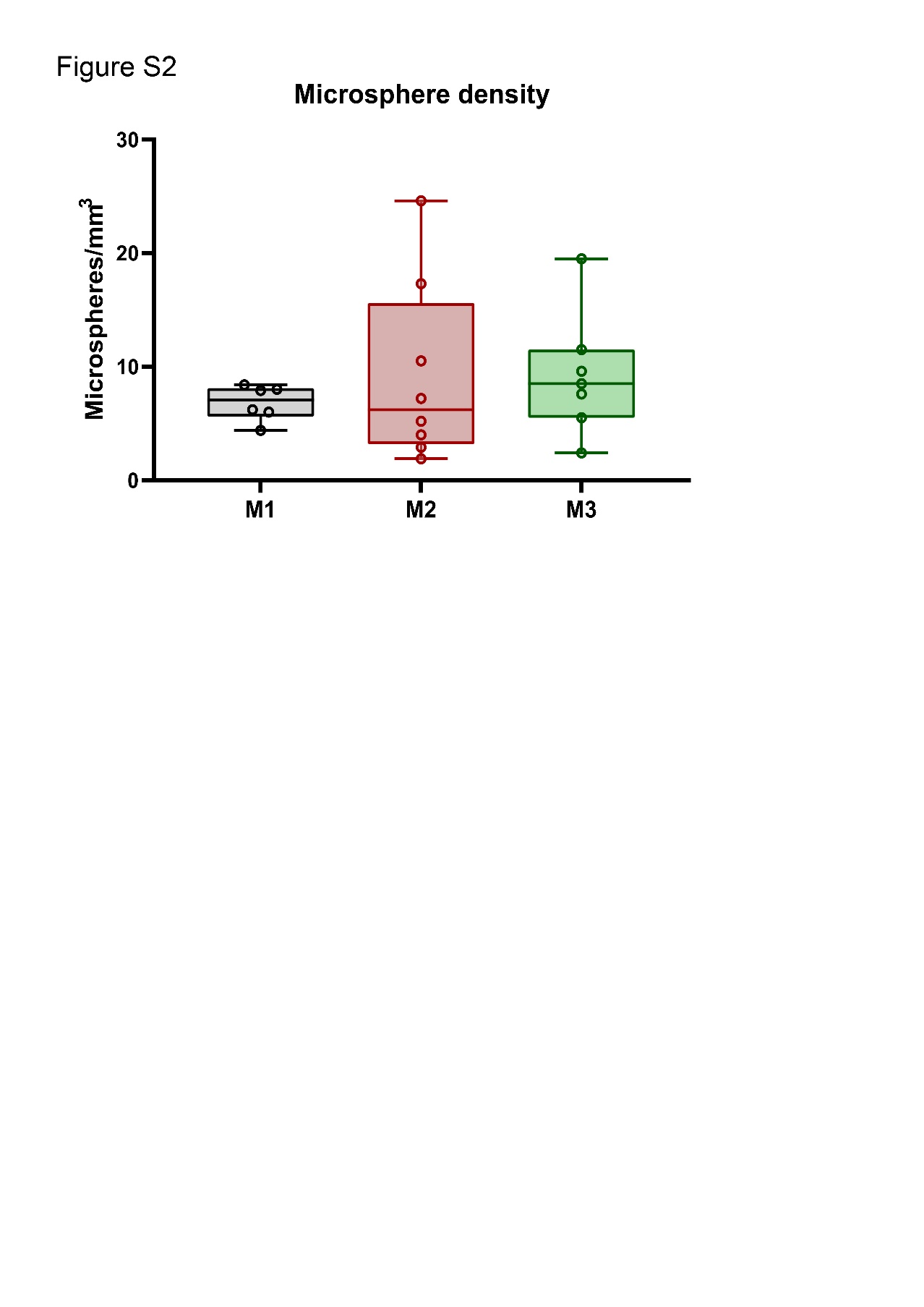


*Figure S2:* Density of deposited microspheres in the brain. Data are depicted as median ± IQR (box) and min – max (whiskers), each data point represents an individual animal (M1: n=6; M2: n=8; M3: n=7). Densities are not significantly different between groups (p=0.69, Kruskal-Wallis test).

*
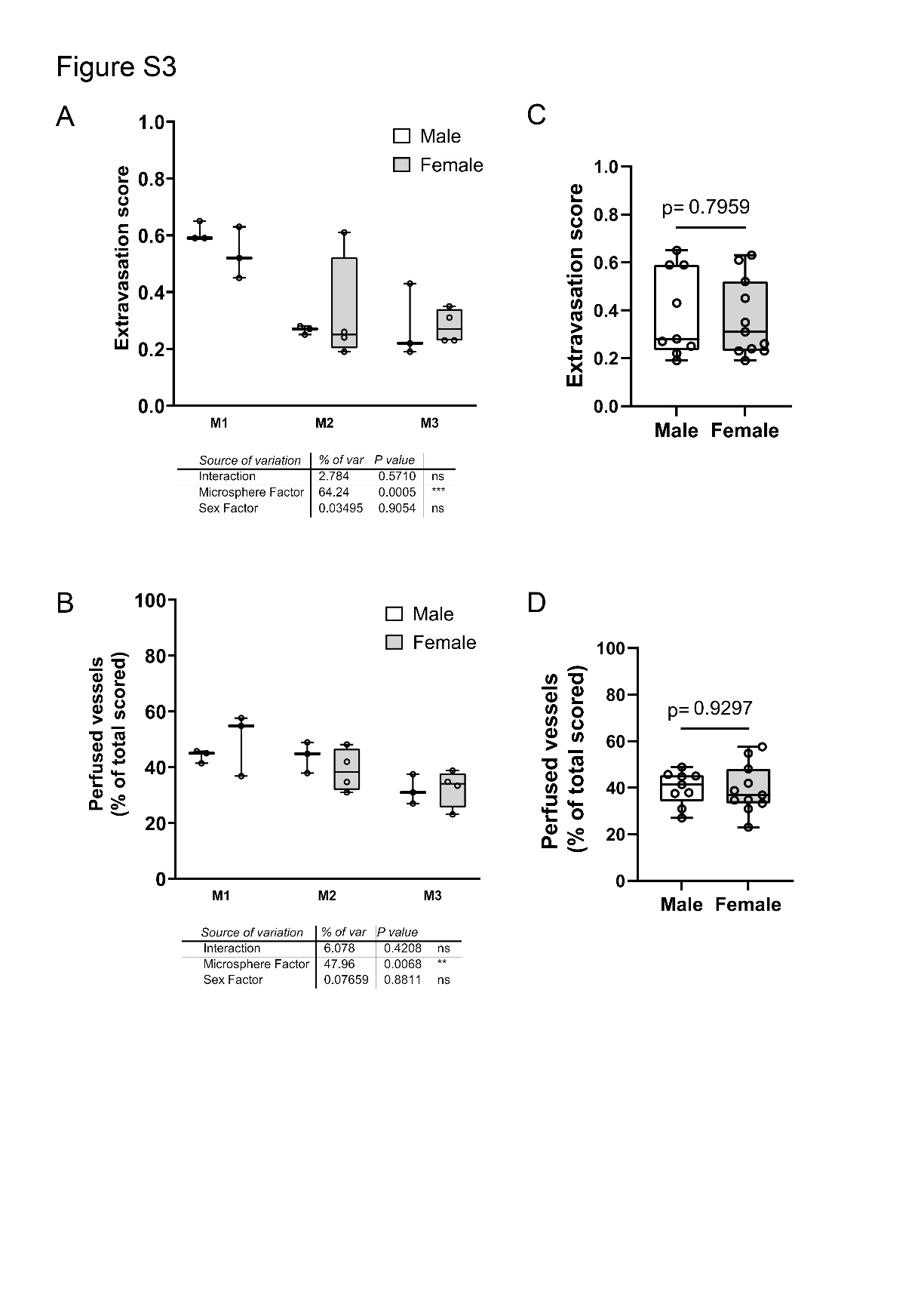
*

*Figure S3.* Sex has no effect on extravasation score and vessel perfusion. Two-way ANOVA was performed for animals stratified by the independent variables “Sex” (male; open boxes, female; filled boxes) and “Microspheres” to assess whether sex affects **A)** extravasation score and **B)** vessel perfusion. Since the groups are rather small when stratified by microsphere class, animals were pooled for microsphere class to assess whether there is a difference in **C)** extravasation score and **D)** vessel perfusion between male and female rats with an unpaired t test. Data are depicted as median ± IQR (box) and min – max (whiskers), each data point represents an individual animal. A and C) Male M1: n=3, female M1: n=3, male M2: n=3, female M2: n=4, male M3: n=3, female M3: n=4. B and D) Male: n=9, female: n=11.


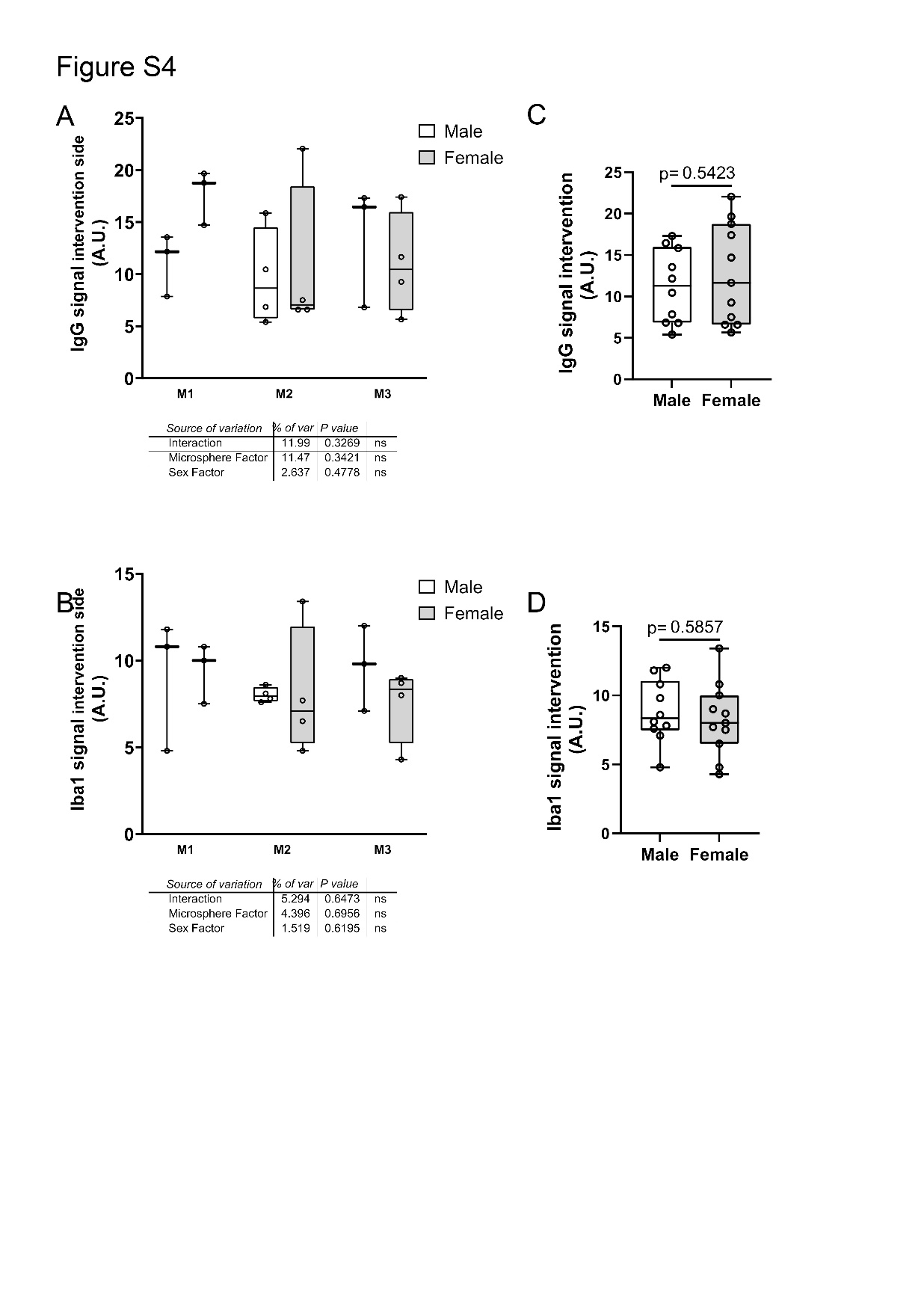


*Figure S4.* Sex has no effect on IgG and Iba1 signal intensity. Two-way ANOVA was performed for animals stratified by the independent variables “Sex” (male; open boxes, female; filled boxes) and “Microspheres” to assess whether sex affects **A)** IgG signal intensity in the intervention side and **B)** Iba1 signal intensity in the intervention side. Since the groups are rather small when stratified by microsphere class, animals were pooled for microsphere class to assess whether there is a difference in **C)** IgG signal intensity in the intervention side and **D)** Iba1 signal intensity in the intervention side between male and female rats with an unpaired t test. Data are depicted as median ± IQR (box) and min – max (whiskers), each data point represents an individual animal. A and C) Male M1: n=3, female M1: n=3, male M2: n=4, female M2: n=4, male M3: n=3, female M3: n=4. B and D) Male: n=10, female: n=11.

Table S1. Absolute numbers scored for extravasation status at day 14

|  | | | | | | |
| --- | --- | --- | --- | --- | --- | --- |
|  | M1 (n=6) | | M2 (n=7) | | M3 (n=7) | |
| In | | 134 | | 201 | | 241 |
| Going out | | 39 | | 50 | | 49 |
| Out | | 41 | | 12 | | 23 |
| TOTAL | | 214 | | 263 | | 313 |

Table S2. Percentage extravasation status at day 14

|  | | | | |
| --- | --- | --- | --- | --- |
|  | M1 (n=6) | | M2 (n=7) | M3 (n=7) |
| In | | 62.8 (60.6-64.8)* | 76.7 (75.0-78.6) | 83.3 (71.4-84.4)* |
| Going out | | 14.1 (12.7-25.5) | 19.6 (19.1-25.0) | 13.5 (9.4-19.6) |
| Out | | 20.7 (14.8-25.2)** | 3.3 (2.4-4.3)** | 6.3 (5.9-7.5) |

Data is depicted as median (IQR). *P=0.0134, M1 vs M3; **P=0.0021, M1 vs M2; Kruskal-Wallis with Dunn’s multiple comparisons test

Table S3: Perfusion of vessels related to extravasation status of microspheres at Day 14

| M1 (n=6) | Perfused (#) | Nonperfused(#) | Total scored (#) | Perfused (%) | p-values (χ^2^) |
| --- | --- | --- | --- | --- | --- |
| In | 22 | 105 | 127 | 17.3 | <0.0001 |
| Going out | 35 | 5 | 40 | 87.5 |  |
| Out | 38 | 0 | 38 | 100 |  |

| M2 (n=7) | Perfused (#) | Nonperfused(#) | Total scored (#) | Perfused (%) | p-values (χ^2^) |
| --- | --- | --- | --- | --- | --- |
| In | 45 | 147 | 192 | 23.4 | <0.0001 |
| Going out | 46 | 3 | 49 | 93.9 |  |
| Out | 11 | 0 | 11 | 100 |  |

| M3 (n=7) | Perfused (#) | Nonperfused(#) | Total scored (#) | Perfused (%) | p-values (χ^2^) |
| --- | --- | --- | --- | --- | --- |
| In | 35 | 196 | 231 | 15.2 | <0.0001 |
| Going out | 43 | 5 | 48 | 89.6 |  |
| Out | 19 | 3 | 22 | 86.4 |  |
